# F171. ALTERED DIFFUSIVITY IN THE BRAIN OF PATIENTS WITH SCHIZOPHRENIA: A DIFFUSION WEIGHTED MAGNETIC RESONANCE IMAGING STUDIES WITH PUBLIC NEUROIMAGING DATA

**DOI:** 10.1093/schbul/sby017.702

**Published:** 2018-04-01

**Authors:** Young Tak Jo, Yeon Ho Joo, Harin Kim, Seung-hyun Shon, Woon Yoon, Sung Woo Joo, Jungsun Lee

**Affiliations:** 1 Asan Medical Center, University of Ulsan College of Medicine; 2 Korean Armed Forces Capital Hospital; 3 Republic of Korea Marine Corps

## Abstract

**Background:**

In recent decades, numerous in vivo brain imaging studies utilizing diffusion weighted MRI (dMRI) technique have focused on altered diffusivity in brains of patients with schizophrenia. However, the literature has not reached at consistent consensus despite a few interesting and promising results. In this study, we investigated whether or not various measures of dMRI (FA, AD, RD, and TR) are altered in patients with schizophrenia by comparing them in both patients and healthy controls with public neuroimaging data from SchizConnect (http://schizconnect.org).

**Methods:**

The final data set was consisted of 121 schizophrenia patients and 119 healthy controls. After verifying 161 anatomical regions of interest (ROIs), we estimated the mean value and standard deviation of fractional anisotropy (FA), axial diffusivity (AD), radial diffusivity (RD), and trace (TR) in each ROI among the healthy controls. After that, we calculated the Z-score of each single ROI in every individual brain of both patients and healthy controls. The Z-score information of each person is then integrated into two location-independent measures. One is the total number of “abnormal” lesions, in which the absolute Z-score is above the cut-off value estimated by the Bonferroni correction, and the other is the largest absolute Z-score. After all, by using Welch two-sample t-test, we compared these two measures between the groups of patients and healthy controls.

**Results:**

The number of abnormal lesions was notably increased in patients group, in terms of RD (p=0.01063) and TR (p=0.009329). Meanwhile, no statistically significant differences related to FA and AD were observed. On the other hand, it was found that the largest absolute Z-score was elevated in patients group, in terms of AD (p=0.03371), RD (p=0.0001762), and TR (p<0.00001). Otherwise, no significant differences related to FA were observed.

**Discussion:**

In this study, we found a few remarkable differences of familiar measures, especially TR, between brains of patients with schizophrenia and healthy controls. This suggests that there should be some subtle changes in the brains of patients with schizophrenia, including microstructural destruction.

